# Understanding secondary hypogammaglobulinemia and its implications for cancer prognosis in children: A retrospective cohort study

**DOI:** 10.7705/biomedica.7584

**Published:** 2024-12-23

**Authors:** Ana Lucía Guzmán, Isabella Villamil, Sofia Martinez-Betancur, Oriana Arias- Valderrama, Jacobo Triviño-Arias, Jessica Largo, Viviana Lotero, Alexis Franco, Ximena Castro, Pamela Rodríguez, Luz Ángela Urcuqui, Diego Medina, Manuela Olaya

**Affiliations:** 1 Departamento de Medicina, Facultad de Salud, Universidad ICESI, Cali, Colombia Universidad ICESI Departamento de Medicina Facultad de Salud Universidad ICESI Cali Colombia; 2 Centro de Investigaciones Clínicas, Fundación Valle del Lili, Cali, Colombia Fundación Valle del Lili Centro de Investigaciones Clínicas Fundación Valle del Lili Cali Colombia; 3 Departamento de Alergología Pediátrica, Fundación Valle del Lili, Cali, Colombia Fundación Valle del Lili Departamento de Alergología Pediátrica Fundación Valle del Lili Cali Colombia; 4 Departamento de Hemato-oncología Pediátrica, Fundación Valle del Lili, Cali, Colombia Fundación Valle del Lili Departamento de Hemato-oncología Pediátrica Fundación Valle del Lili Cali Colombia

**Keywords:** Agammaglobulinemia, neoplasms, pediatrics, child., agammaglobulinemia, neoplasias, pediatría, niño.

## Abstract

**Introduction.:**

Immunodeficiencies are disturbances in the immune system that can affect cell function, quantity, or both. They can be either primary, associated with genetic defects, or secondary, linked to external factors such as hemato-oncological conditions. Secondary immunodeficiencies can lead to the initiation, reactivation, or acceleration of latent, residual, or active infections, which are the leading cause of mortality.

**Objective.:**

To elucidate the occurrence and clinical characteristics of hypogammaglobulinemia in pediatric oncology patients in a high-complexity hospital in Colombia between January 2020 and December 2022.

**Materials and methods.:**

We conducted an observational study with patients under 18 years old with a cancer diagnosis, serum immunoglobulins measurements at the time of the diagnosis, and later follow-up during treatment.

**Results.:**

We included 133 patients with a median age of eight years. Based on local guidelines of immunoglobulin levels for age, all patients had normal values at the time of cancer diagnosis. In the follow-up, the most significant reduction among all ages was for IgA and was related to infections and death.

**Conclusions.:**

Our findings highlight the importance of measuring immunoglobulin levels at the time of the cancer diagnosis, as hypogammaglobulinemia may be linked to a poorer prognosis. Early detection could potentially improve patient outcomes.

## Resumen

**Introducción.:**

Las inmunodeficiencias son alteraciones del sistema inmunológico, que pueden afectar la función, el número de células o ambas. Pueden ser primarias, asociadas con defectos genéticos, o secundarias, vinculadas a factores externos como condiciones hemato-oncológicas. Estas inmunodeficiencias secundarias pueden llevar al inicio, reactivación o aceleración de infecciones latentes, residuales u oportunistas, que son la principal causa de mortalidad de estos pacientes.

**Objetivo.:**

Dilucidar la ocurrencia y las características clínicas de la hipogammaglobulinemia en pacientes pediátricos oncológicos atendidos en un hospital de alta complejidad de Colombia entre enero de 2020 y diciembre de 2022.

**Materiales y métodos.:**

Se realizó un estudio observacional de pacientes menores de 18 años con diagnóstico de cáncer, medición de inmunoglobulinas en el momento del diagnóstico y seguimiento durante el tratamiento.

**Resultados.:**

Se incluyeron 133 pacientes con una edad media de ocho años. De acuerdo con las guías locales para la edad, se encontró que todos los pacientes tenían valores normales de inmunoglobulinas. En el seguimiento, la reducción más significativa en todas las edades fue la de IgA y se relacionó con la presencia de infecciones oportunistas y mortalidad.

**Conclusiones.:**

Los resultados de este estudio destacan la importancia de medir las inmunoglobulinas en el momento del diagnóstico de cáncer, ya que la hipogammaglobulinemia podría estar vinculada con un pronóstico desfavorable. La detección temprana de hipogammaglobulinemia podría estar vinculada a un pronóstico desfavorable.

Immunodeficiencies are disturbances in the immune system that can affect cell function, quantity, or both. They can be either primary, associated with genetic defects, or secondary, linked to external factors such as hemato-oncological conditions and their respective treatments. Primary immunodeficiencies encompass a wide range of inherited disorders, each with unique clinical manifestations. Among the various causes of secondary immunodeficiencies, hematological-oncological diseases, and their treatments stand out as significant contributors ^1^^,^^2^.

Cancer constitutes one of the leading causes of mortality in childhood and adolescence, with its prevalence and incidence increasing worldwide. According to the World Health Organization (2021), approximately 280,000 children and adolescents aged 0 to 19 are diagnosed with cancer annually ^3^. High-income countries exhibit a mortality rate of less than 20%, while in low-income or middle-income countries, the mortality rate can exceed 70% ^3^.

Cancer and immunodeficiencies share a common feature: the failure or absence of various components of the immune system ^4^^,^^5^. The close relationship between these conditions makes their co-occurrence particularly concerning, as it heightens the predisposition to several complications ^4^^,^^6^. These complications include the initiation, reactivation, or acceleration of latent, residual, or active infections, which are the primary cause behind increased mortality rates ^1^^,^^7^^-^^9^

Immunoglobulin replacement therapy for infection prevention is well-established and supported by a wealth of clinical data in primary immunodeficiencies ^10^. In contrast, there is a noticeable dearth of evidence concerning the challenges associated with immunoglobulin replacement therapy in secondary immunodeficiencies ^4^. Most published guidelines in this area are extrapolated from the experience with adult primary and secondary immunodeficiencies ^7^.

This study aimed to provide a comprehensive description of the occurrence and clinical characteristics of hypogammaglobulinemia in pediatric patients with an oncological diagnosis at *Fundación Valle del Lili* from January 2020 to December 2022.

## Materials and methods

We conducted a retrospective cohort study from January 2020 to December 2022. We included patients with cancer diagnoses treated at the *Fundación Valle del Lili* in Cali, Colombia. The patients were identified in the hospital database using the hospital registry of children’s cancer. The inclusion criteria were patients between 0 and 18 years of age who received treatment in the hospital for cancer and whose serum immunoglobulins were measured at the time of the cancer diagnosis. We excluded those who had a diagnosis of hypogammaglobulinemia before the diagnosis of cancer, pregnant patients, and patients with autoimmune diseases.

We collected demographic information, such as age, diagnoses, immunoglobulin levels at diagnosis and 3, 6,12, and 18 months later, and variables related to infections or multidrug resistance. The outcome of death was also collected. However, we do not have data on the number of febrile neutropenia episodes or hospital stay days.

The analyses were performed with the clinical characteristics of patients. The distribution of the quantitative variables was calculated using the Shapiro-Wilk test. We used a two-sided chi-squared test to evaluate the differences between groups; a p value less than 0.05 was considered statistically significant. The software used for the analysis was R Studio, version 2022.07.2.

### Ethical considerations

Ethical approval was waived by the institutional review board (*Comité de Ética en Investigación Biomédica of Fundación Valle del Lili*), with the act number 1961.

## Results

A sample of 165 patients was selected from the pediatric oncology database of Fundación Valle del Lili and 133 met the inclusion criteria. The median age was eight years (IQR = 4-13). The diagnosis of lymphoblastic acute leukemia was the most common in 85 patients (64%), followed by Hodgkin’s lymphoma in 22 (16.5%) (table 1).

**Table 1.** Diagnoses of the patients included in the pediatric oncology population registries of the *Fundación Valle del Lili*, Cali, Colombia, according to the International Classification of Diseases (tenth revision) (N = 133)

International Classification of Diseases	n	(%)
Acute lymphoblastic leukemia	85	(64)
Hodgkin's lymphomas	22	(16.5)
Acute myeloid leukemias	12	(9)
Burkitt lymphoma	7	(5.3)
Chronic myeloid leukemia	2	(1.5)
Non-Hodgkin's lymphomas	3	(2.2)
Lymphoblastic lymphoma (diffuse)	2	(1.5)

**Figure 1.**
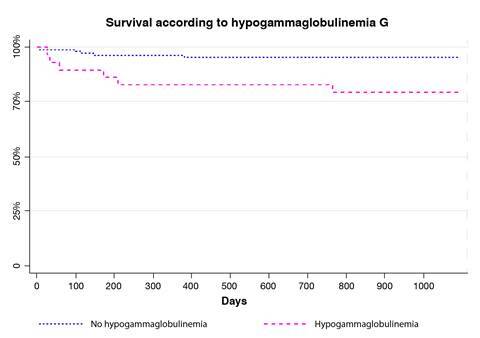
Survival according to hypogammaglobulinemia G

**Figure 2.**
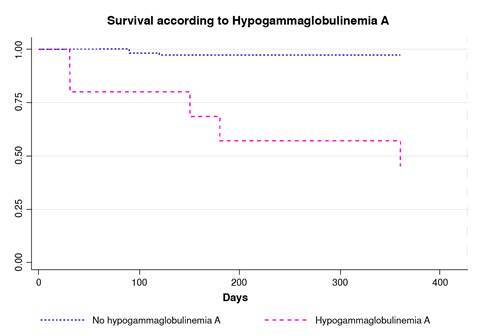
Survival according to hypogammaglobulinemia A

The immunoglobulin levels at diagnosis, according to age and local guidelines, are shown in table 2. Immunoglobulin monitoring revealed that not all patients had measurements as stipulated in the methodology. Nevertheless, the available data indicated that none of the patients had hypogammaglobulinemia of any isotype at diagnosis, and a significant decline in IgA levels was observed during follow-up, particularly among those who died. However, a substantial number of patients lacked complete immunoglobulin data, limiting the analysis. Patients diagnosed with hypogammaglobulinemia A or G at any stage of treatment exhibited a reduced survival rate during the evaluated period (figures 1 and 2).

**Table 2.** Median immunoglobulin levels at the moment of diagnosis by age groups

Immunoglobulin	1 year (n = 9) Median (IQR)	2-6 years (n = 44) Median (IQR)	7-12 years (n = 42) Median (IQR)	Over 12 (n = 38) Median (IQR)	value^1^
A	52 (37-94)	91 (65-115)	109 (70- 149)	150 (89-229)	0.004
M	45 (34-67)	74 (48-106)	69 (46- 104)	86 (64- 112)	0.2
G	837(640- 1,056)	888(748- 1,100)	988 (834- 1,163)	1,247(877- 1,498)	0.017

IQR: interquartile range

^1^ Kruskal-Wallis rank sum test

One hundred sixteen (87%) patients presented infections during the treatment for their oncological pathology; 50 (43%) of these had an infection by an opportunistic agent. All patients with hypogammaglobulinemia G (according to international tables) had an infection at some point during their follow-up (table 3).

**Table 3.** Hypogammaglobulinemia by age associated with infections (n = 29)

Hypogammaglobulinemia	n	(%)
1 year (n = 9)	5	(17)
2-6 years (n = 44)	5	(17)
7-12 years (n = 42)	9	(31)
Over 12 years (n = 38)	10	(34)

Only 11 patients died during the study period: seven (63%) had infections caused by conventional encapsulated bacteria such as *Haemophilus influenzae, Streptococcus pneumoniae*, spp., *Neisseria meningitidis, Pseudomonas* spp. and four (36%) by atypical bacterial pathogens such as *Mycoplasma pneumoniae* and *Chlamydia pneumoniae*. Of the deceased patients, five (45%) had hypogammaglobulinemia primarily affecting IgA levels. Within the latter, three (60%) had infections caused by conventional pathogens, and two (40%) by atypical pathogens.

## Discussion

Immunodeficiencies are disturbances of the immune system and can be primary or secondary. Cancer and immunodeficiencies share the failure of various immune components ^4^^,^^5^. The close relationship between these conditions makes their co-occurrence particularly concerning. ^4^^,^^6^.

In this study, the most prevalent diagnosis was acute lymphoblastic leukemia in 85 (64%) patients, followed by Hodgkin's lymphomas in 22 (16.5%), consistent with the findings of a literature review conducted by Sánchez *et al.*^4^. All patients with lymphoma did not have primary immunodeficiency. According to the review of global literature, lymphoma has been observed as a comorbidity in approximately 16% of patients with primary immunodeficiencies. This finding is supported by a study based on the cancer incidence in the United States Immune Deficiency Network Registry, which revealed a significantly elevated risk of developing cancer in this population. Notably, this risk increases up to tenfold in the specific case of lymphoma in patients with primary immunodeficiencies. ^3^^,^^11^

Infections are particularly relevant in the population with secondary hypogammaglobulinemia. It currently represents the primary cause of morbidity and mortality in these patients, with a burden of up to 25 - 50% of the mortality rate, according to global literature ^4^^,^^11^.

Table 2 presents median values of IgG, IgM, and IgA based on age; in all cases, levels were normal at the time of cancer diagnosis, contradicting some literature findings associating secondary immunodeficiencies with systemic disorders, medications, and critical or chronic illnesses ^4^. Further studies with larger populations and extended follow-up periods are needed to validate these observations and achieve serial sampling of immunoglobulins in routine paraclinical examinations of children with cancer.

Our results indicate a higher mortality rate in individuals with lower IgA values, potentially due to infections, which were present in 7 of 11 patients who died. Despite the absence of standardized measurement times, the study revealed a crucial and unexpected finding: Lower IgA levels in pediatric patients with an oncological diagnosis could be associated with disease progression, compared to surviving patients with stable IgA levels. Considering that a significant percentage of patients in our cohort presented with hypogammaglobulinemia A, which has been linked to an increased incidence of infections, it is reasonable to infer that these patients might be at a higher risk of morbidity and mortality. However, due to the lack of follow-up immunoglobulin A values in our patients, we could not draw a conclusion. This limitation underscores the need for future research to address this knowledge gap.

Overall survival was not affected by the diagnosis of hypogammaglobulinemia G at any time of the treatment according to the reference intervals for immunoglobulins in the pediatric population provided by Jolliff *et al.*^12^. However, all patients diagnosed with hypogammaglobulinemia G at the beginning of their monitoring developed infections at some point during the observation period. These findings support global literature by highlighting a clear association between hypogammaglobulinemia G and an increased risk of infections. The results presented in table 3 provide a detailed insight into this relationship, consolidating evidence of the clinical significance of hypogammaglobulinemia G in the pediatric oncological context.

Regrettably, during data collection, it became evident that immunoglobulin measurement times were not standardized. Consequently, follow-up assessments were conducted randomly rather than at regular intervals (*e.g*., 6 or 12 months or at the end of treatment), preventing an organized interpretation of data according to the evolution and follow-up time.

General measures for patients with secondary immunodeficiencies include strategies to minimize exposure to infections, antibiotic prophylaxis, and immunoglobulin replacement, all of which should be standardized through national or international protocols ^5^. Pediatric patients with oncological conditions should undergo monitoring of serum immunoglobulin levels from the time of diagnosis to warrant early detection of secondary immunodeficiencies.

Early diagnosis can lead to the timely administration of immunoglobulin replacement treatment, potentially reducing morbidity and mortality. Enhancing adherence to protocols and management guidelines by healthcare professionals caring for these patients is crucial ^4^. These measures can significantly contribute to more effective patient management and offer recommendations to mitigate the risk of frequent or severe infections ^5^.

This study serves as an essential starting point, providing key guidance for conducting prospective studies with a larger patient cohort by establishing protocols and groundwork for the implementation of standardized serial determinations to decrease mortality rates in the pediatric oncology population.
